# Dentists’ entrepreneurial intention and associated factors in public hospitals in major cities in Guangdong (South China): a cross-sectional study

**DOI:** 10.1186/s12903-020-01331-z

**Published:** 2020-11-23

**Authors:** Jiabi Wang, Bin Peng, Hongzhi Zhou, Jing Hua Zhang

**Affiliations:** 1 School of Business, Macau University of Science and Technology, Avenida Wai Long, Taipa, Macao S.A.R. China; 2 Nokia Solutions and Networks System Technology (Beijing) Co., Ltd., No. 1, Wangjing East Road, Chaoyang District, Beijing, 100101 China

**Keywords:** Entrepreneurial intention, Dentist career, Public hospitals in china

## Abstract

**Background:**

A rapid growth in private dentistry in China has been observed during recent years. Promoting the entrepreneurship of dentists has increasingly received attention in both dentistry and dental education worldwide. However, understanding about the unique features of entrepreneurial behaviors of dentists remains inadequate.

**Methods:**

This study examines dentist’s entrepreneurial intention (EI), which was represented by his/her intention of leaving the public hospital system to be engaged in the private sector. Through a snowball sampling method, a total of 336 questionnaires from public hospitals in five major cities in Guangdong Province (China) were collected. The association between the dentists’ EI and their individual characteristics were analyzed using a logistic regression model.

**Results:**

In the sample studied, 35.7% of the respondents reported to have EI. Female dentists are less likely to report EI (OR = 0.365, *p* = 0.001). Dentists in the age group of 36 to 45 years (OR = 14.205, *p* = 0.012) and those aged over 45 years (OR = 8.45, *p* = 0.066) reported respectively a much stronger EI than those in their 20s. Compared with intern dentists, attending dentists (OR = 7.812, *p* = 0.016) and associate/chief dentists (OR = 9.857, *p* = 0.021) were significantly more likely to report EI. Those with master level (OR = 0.221, *p* = 0.021) or doctorate degrees (OR = 0.118, *p* = 0.005) are much less likely to report EI. Meanwhile, those in mid-large hospitals (with 101–200 employees) (OR = 3.554, *p* = 0.036) and small hospitals (with < 50 employees) (OR = 2.398, *p* = 0.044) reported a stronger EI than those in large hospitals. Additionally, dentists’ entrepreneurial behaviors, risk aversion attitudes and their family background all have significant associations.

**Conclusions:**

Since dentistry is a knowledge-intensive industry, dentists’ entrepreneurial behaviors have their own features. The findings by this study suggest that, accumulation of practical skills in a dental career, as implied by age, professional qualifications and leadership skills, help to promote EI, whereas an academic oriented education degree per se does not. Dentists in mid-large and small hospitals, rather than in top large hospitals in China, have higher EI. Additionally, female dentists may need more social supports to develop a higher EI. These findings have practical implications for the promotion of EI among dentists.

## Background

### Dentists’ entrepreneurial activities and intention

Dentists traditionally have a higher level of entrepreneurship*,* or are more likely than other healthcare professionals (such as physicians, surgeons or nurses), to start up their own practice, or move into private practice [1, 2]. Entrepreneurship refers to the ability to identify new opportunities, the possession of executive skills to obtain resources and implement plans, as well as leadership skills, including risk attitude and tolerance [3, 4]. Specifically, the concept of entrepreneurship in dentistry involves initiating or developing a dental practice or relevant business, organizing the necessary financial and human resources, and taking the associated risks and rewards [4]. Dentists may also be engaged in the manufacturing of dental instruments, equipment or materials. During the past decades, as results of healthcare reforms worldwide, market factors (such as service competition and patient satisfaction) are playing an increasingly important role in the operation environment of dentistry. Promoting the entrepreneurship of dentists has increasingly received attention in both dentistry and dental education worldwide [1, 4, 5]. To achieve this goal effectively, it is necessary to have an in-depth understanding of dentist’ entrepreneurial intention (EI) and related influential factors.

Entrepreneurial intention (EI) is defined as “the conscious state of mind that precedes action and directs attention toward entrepreneurial behaviors such as starting a new business and becoming an entrepreneur” [6, 7]. As the first stage of a series of actions in organizational founding, EI is an important part of the business planning process [8, 9], reflecting an individual’s readiness to engage in subsequent entrepreneurial activities [10–14]. It may also be influenced by factors, such as an entrepreneur’s demographic, professional knowledge, technical skills, entrepreneurial behaviors [15], as well as psychological characteristics [16, 17]. Employees in knowledge–intensive industries may demonstrate entrepreneurial intention and behaviors with some unique features [16–18]. For example, in general, business entrepreneurs are more likely to be active with start-up and innovations in their 20s [19–21], whereas dentists’ optimal time for starting a private practice may be postponed due to education and professional qualification requirements. Other factors, such as a family business background or the social-cultural environment, may also influence an individual’s EI through the channels of social skills development, confidence and business values [22, 23].

### The demand and supply of dental care service in China

Due to traditional ignorance about oral health and past social-economic reasons, the general oral health status of Chinese residents is a serious concern and there has been a rapid growth in demand for dental service [24]. In 2017, the caries incidence of the population was greater than 50%, and less than 50% of the residents had a healthy periodontal condition [25]. The caries incidence for children at age of 5 years was 70.9%, and 34.5% at the age of 12 years [25]. For adults between the age of 35 and 44 years, the dental cavity detection frequency was 96.7% and the gingival bleeding detection frequency was 87.4% [25]. With the significantly enhanced awareness of preventive dental health and improved living standards in China during the past decade, public demand for dental care surged quickly with an annual growth rate of about 11% [26]. In 2020, China's dental care market was estimated to be worth RMB100 billion (US$14 billion) [26].

The current social health insurance scheme in China only covers minimum basic treatment-oriented dental services and does not include prevention-oriented dental care services, dental crowns with advanced materials, orthodontics or dental implants [24]. It is estimated that over 85% of the dental costs have to be paid out of pocket [24]. Many urban middle-class residents, especially those in central cities, prefer to choose private dental clinics because they are willing to and can afford to pay for the better patient experience provided by private dental clinics [27, 28]. These private clinics can provide a facility with a patient-friendly environment, patient communications with patients, advanced diagnostic and treatment equipment, a short or no waiting time, flexibility in appointments, and so on [26, 29, 30].

At the current stage, the dental care supply in China is still insufficient to meet the rising demand. By 2018, there were 314,347 trained dentistry personnel in mainland China, among whom 171,587 (54.6%) were dentists, 37.8% were dental nurses and 7.6% were dental technicians. The density of dentistry personnel (per 1000 population) was about 0.129 in 2018, while the WHO standard is 0.2 [31]. There were in total 75,399 dental service units among all healthcare providers in mainland China [31]. By institution number, 73.8% of these service units exist as dental departments of general hospitals [24].

To improve the supply of dental health care, over the past five years there has been some policy reforms in China, such as multi-sited practicing by qualified dentists and a simplified process of private clinic license application and approval. Significantly, there are favorable policies encouraging private venture capital to enter the dentistry field in China [24, 32]. Due to these reasons, investment capital has actively flowed into the private dental sector and some large oral-care companies have tried to establish chain clinics through the acquisition of existing stand-along clinics, especially those owned and operated by qualified dentists (for example, dentists who graduated from leading dental schools or who are working in top public hospitals in the region) [26, 29, 33]. The rapid growth of the private dental care market in China has created a high demand for qualified dentists with entrepreneurship from public hospitals, where most dentists practice and develop in their sophistication of practice [26]. While a career in public hospitals may provide job security and a decent professional reputation, private practice and dental entrepreneurs have the advantages of a more attractive income and the potential of a lucrative financial return from a successful dental venture business.

As employees in a knowledge–intensive industry, dentists’ entrepreneurial activities may have some unique features and associated factors. However, currently there is only a very small volume of literature studying this aspect of dentists [5, 34, 35]. Some studies focused on dental students [36–40], but they are not yet perfectly comparable to dentists in their actual career path with various social-economic responsibilities. The recent rapid growth of private dentistry in China and the high demand for dentists in public hospitals has provided a good research opportunity.

### Study location

This study focuses on the dentists in public hospitals in five major cities of Guangdong Province (Guangzhou, Shenzhen, Foshan, Zhuhai, and Zhongshan). Located in the Pearl River Delta Area in Guangdong province, Guangzhou and Shenzhen are among the top megacities of China and the studied cities are among the most economically developed areas in China with a high level of personal income. Since the private economy (especially small to medium enterprises) developed early in Guangdong across all industries, including healthcare, residents in this area accept private dental care that meets their quality and personal experience preference [26]. In industrial cities in Guangdong, such as Shenzhen, with limited public medical resources and a strong individual capacity to pay, the private dental sector has very large potential market growth opportunity [26].

In 2018, there were 20,681 certified dentistry personnel in Guangdong province [41]. The density of dentistry personnel (per 1000 population) was about 0.18, whereas the national average level was 0.129. There are in total 172 general public hospitals and specialized dental hospitals in Guangdong Province. There are 104 hospitals (60%) located in the 5 major cities selected in this study while the remaining 17 cities in total have 68 hospitals (40%) [41]. Specifically, 53 hospitals (30%) are located in Guangzhou City (the capital of Guangdong Province), 21 (12%) in Foshan, 17 (10%) in Shenzhen, 8 (5%) in Zhuhai and 5 (3%) in Zhongshan [26].

### Aim of this study

This study aims to examine the entrepreneurial intentions of dentists in the public hospitals in major cities of Guangdong Province, China, and the associated key factors, including educational background, professional qualification, years of practice, their entrepreneurial or leadership skills, family background, as well as risk aversion level.

## Methods

### Ethics approval and consent to participate

Ethical approval of this study was obtained from the Research Ethics Committee at the School of Business, Macau University of Science and Technology (No: MUST/MSB/D/19/0128). The verbal informed consent procedure was approved by the Research Ethics Committee. The questionnaire has a brief cover letter in written form, describing the research purpose of the collected information and the confidential guarantee policy. Before starting the questionnaire, the ethics statement was disclosed to all respondents and verbal informed consents were obtained from all respondents. All responses were voluntary and anonymous.

### Questionnaire

The questionnaire adopted an established scale to measure the key concepts of entrepreneurial intention and behaviors [42] and was pre-tested for validity by collegiate faculty, dentists, and dental students randomly selected from major public hospitals in Guangzhou, China. The reliability of the scales was estimated to be 0.81. (See Additional file 1 for the full version of the questionnaire.)

### Sampling

A Snowball sampling method was adopted. Two or three senior dentists in the major public hospitals in each city were first randomly sampled, then each of them gave multiple referrals to other qualified dentists in their cities. This pattern was repeated until a sufficient number of dentists became available for the sample. The number of dentist respondents is proportional to the dental healthcare workforce population in each city [26, 41]. About 51% of the respondents in this sample are from Guangzhou, 20% from Foshan, 16% from Shenzhen, 8% from Zhuhai and 5% from Zhongshan.

The sampling and survey process was performed during the ten months between September 2018 and June 2019. A total of 400 questionnaires were distributed in either paper or electronic forms through WeChat (the dominant social media App in China), depending on the respondents’ preference and convenience. Eventually, 336 valid responses were received (effective return rate = 84%), equivalent to about 3.6% of the total registered dentist population in the studied area. All questionnaires were self-administrated. All data collected through the questionnaires were entered and coded.

## Statistical methods

### Dependent variable

EI is a useful and practical approach to understanding actual entrepreneurial behavior. This survey of dentists’ EI captures their objective orientation, such as plans, wishes and preference for engaging in entrepreneurial dental business activities [43].

It was measured by one single question adapted from Krueger et al. [44], “It is likely that I will start and run my business in dentistry in the near future.” Responses were measured with a 5-point Likert scale, with 1 being least likely and 5 being most likely. For logistic regression analysis, a dummy variable of EI was then created according to the number values of the answers. EI was assigned with the value of “1” if answers were “5 = most likely”, or “4 = likely”. EI took the value of “0” for other answers (3 = neutral, 2 = unlikely, 1 = very unlikely).

### Independent variables

This study hence includes these relevant factors accordingly.

#### Demographic and professional characteristics

This part contains questions about gender, age, educational level, professional qualification and years of practice. The hospital workplace was measured in terms of employee numbers, rather than bed numbers, because most dental treatment is performed with outpatients, rather than inpatients.

#### Entrepreneurial behavior

This variable reflects personalities and skills associated with identifying and exploring business opportunities, taking risks and being engaged in implementation of business plans [15]. The scale for measuring entrepreneurial behaviors in this study was adopted from Afsar et al*.* [42], that is especially designed for employee’s entrepreneurial behavior in knowledge–intensive industries [16–18]. Accordingly, the level of a dentist’s entrepreneurial behavior is the average score of six dimensions. (1) “Encourage team members to take the initiative with their own ideas.”; (2) “Inspire team members to think about their work in innovative perspectives”; (3) “Devote time to help team members improve the products and services”; (4) “Vividly describe how things could be in the future and what is needed to reach the goal”; (5) “Effectively organize team to meet a challenge”; (6) “Create an environment where people get excited about making improvement”. All measurements are based on a 5-point Likert scale from 1 (disagree strongly) to 5 (agree strongly).

#### Risk aversion

An individual’s risk-taking attitude may have a direct and significant effect on EI [45]. Strong perceptions regarding the potential loss of business, would lead to a lower level of EI [46]. Risk aversion in this study was measured by the established tool developed by Barbosa et al.[47]. Presented with the statement, “The failure of starting a private business will have a negative impact on my future career.”, respondents were asked to rate how closely this statement reflected their perceptions and responses using a 5-point Likert scale from 1 (disagree strongly) to 5 (agree strongly).

#### Entrepreneurial family background

Family background and parental role models can affect EI through attitudes [48, 49], innovation, and creative capacities [50, 51]. There is a question asked about whether or not a respondent had family/relative members who engaged in entrepreneurship.

### Statistical analysis

The baseline model was a multivariate logistic regression model analyzing the major factors associated with the dependent variable, the dentists’ EI, that is a dummy variable, taking the value of “1” if answering “having entrepreneurial intention”. The independent variables included basic demographic and professional characteristics of the dentists.

The full model added additional variables to capture potential influences from the dentist’s personality, personal skills and resources, such as ‘*entrepreneurial behavior’,* ‘r*isk aversion’* and ‘*entrepreneurial family background’.* All statistical analyses were performed with STATA (version 15).

## Results

Table 1 reports the demographic and professional characteristics of the sample. The result showed that 35.71% of the dentist respondents have EI. About 70.83% of the respondents were female. More than three fourths of the respondents were less than 35 years old and only 11.01% were aged 46 years or more. As for educational level, nearly half of the dentists only have an associate or bachelor’s degree and only 10.71% of them have a doctoral degree. The respondents in the sample hold various professional positions and work experience groups are well represented. Among all public hospitals in this sample, 72.02% of them are the major ones with more than 200 employees. About 78% of the respondents reported to have family members or relatives running or owning dental clinics.

**Table 1:** Demographic and professional characteristics of the respondents (N = 336)

Variables	N (%)
Entrepreneurial intention	
Yes	120 (35.71)
No	216 (64.29)
Gender	
Male	98 (29.17)
Female	238 (70.83)
Age (years)	
20–25	80 (23.81)
26–35	170 (50.6)
36–45	49 (14.58)
46–60	37 (11.01)
Educational level	
Associate degree	73 (21.73)
Bachelor's degree	94 (27.98)
Master's degree	133 (39.58)
Doctoral degree	36 (10.71)
Years of practice	
1 or less	116 (34.52)
2–5	102 (30.36)
6–10	41 (12.2)
11 or more	77 (22.92)
Professional qualification	
Intern	83 (24.7)
Resident	101 (30.06)
Attending dentist	63 (18.75)
Associate/chief dentists	39 (11.61)
Administration	50 (14.88)
Number of employees at workplace hospital	
50 or less	41 (12.20)
51–100	31 (9.23)
101–200	22 (6.55)
201 or more	242 (72.02)
Entrepreneurial family member	
Yes	262 (77.98)
No	74 (22.02)

Table 2 reports details of the survey questions and descriptive statistics about dentists’ risk aversion and entrepreneurial behavior. The average score of risk perception is 3.14, indicating that the dentists were slightly risk averse. While the mean of the dentists’ entrepreneurial behavior score is 3.62, indicating a moderately active level.

**Table 2:** Descriptive statistics of dentists’ risk aversion and entrepreneurial behaviors (N = 336)

Risk aversion	3.14 (mean)	1.04 (s.d.)
Entrepreneurial behavior score*	3.62 (mean)	0.67 (s.d.)

Risk aversion						
	Strongly Disagree (1)	Disagree (2)	Undecided (3)	Agree (4)	Strongly Agree (5)	*P* value**
Q 1. The failure of starting a private business will have a negative impact on my future career. [N (%)]						
	16 (4.76)	87 (25.89)	100 (29.76)	100 (29.76)	33 (9.82)	< 0.01

Entrepreneurial behavior						
	Strongly Disagree (1)	Disagree (2)	Undecided (3)	Agree (4)	Strongly Agree (5)	*P* value*
Q 1. This employee encourages others to take the initiative for their own ideas. [N (%)]						
	1 (0.3)	6 (1.79)	70 (20.83)	203 (60.42)	56 (16.67)	< 0.01
Q 2. This employee inspires others to think about their work in new and stimulating ways. [N (%)]						
	3 (0.89)	12 (3.57)	84 (25)	171 (50.89)	66 (19.64)	< 0.01
Q 3. This employee devotes time to helping others find ways to improve our products and services. [N (%)]						
	5 (1.49)	21 (6.25)	114 (33.93)	155 (46.13)	41 (12.2)	< 0.01
Q 4. This employee vividly describes how things could be in the future and what is needed to get us there. [N (%)]						
	9 (2.68)	43 (12.8)	151 (44.94)	112 (33.33)	21 (6.25)	< 0.01
Q5. This employee gets people to rally together to meet a challenge. [N (%)]						
	7 (2.08)	27 (8.04)	124 (36.9)	146 (43.45)	32 (9.52)	< 0.01
Q 6. This employee creates an environment where people get excited about making improvements. [N (%)]						
	9 (2.68)	21 (6.25)	109 (32.44)	165 (49.11)	32 (9.52)	< 0.01

*The sample average score of Q1 to Q6

**Chi-squared test

Table 3 analyzes the characteristics of the respondents with or without EI respectively. Male dentists were more likely to report having EI. There were no statistically significant differences among various groups of age, educational level, and years of practice. Dentists in large hospitals (with more than 200 employees) are less likely to report having EI, while those in small hospitals (with less than 50 employees) are most likely to report having EI. Additionally, dentists who have entrepreneurial family members were significantly more likely to report having EI. Meanwhile, dentists with a high level of risk aversion are significantly less likely to report having EI.

**Table 3:** Associations between dentists’ entrepreneurial intention and relevant factors (N = 336)

	Have Entrepreneurial intention (*N* = 120) N (%)	Do not have Entrepreneurial intention (*N* = 216) N (%)	*P* value
Gender			
Male	58 (59.18%)	40 (40.82%)	< 0.0001
Female	62 (26.05%)	176 (73.95%)	
Age (years)			
20–25	30 (37.5%)	50 (62.5%)	0.631
26–35	57 (33.53%)	113 (66.47%)	
36–45	21 (42.86%)	28 (57.14%)	
46–60	12 (32.43%)	25 (67.57%)	
Educational level			
Associate degree	28 (38.36%)	45 (61.64%)	0.932
Bachelor's degree	34 (36.17%)	60 (63.83%)	
Master's degree	45 (33.83%)	88 (66.17%)	
Doctoral degree	13 (36.11%)	23 (63.89%)	
Professional qualification			
Intern	31 (37.35%)	52 (62.65%)	0.065
Resident	32 (31.68%)	69 (68.32%)	
Attending dentist	28 (44.44%)	35 (55.56%)	
Associate/chief dentists	18 (46.15%)	21 (53.85%)	
Administration	11 (22.00%)	39 (78.00%)	
Years of practice			
1 or less	45 (38.79%)	71 (61.21%)	0.588
2–5	33 (32.35%)	69 (67.65%)	
6–10	17 (41.46%)	24 (58.54%)	
11 or more	25 (32.47%)	52 (67.53%)	
Number of employees at workplace hospital			
50 or less	22 (53.66%)	19 (46.34%)	0.041
51–100	9 (29.03%)	22 (70.97%)	
101–200	10 (45.45%)	12 (54.55%)	
201 or more	79 (32.64%)	163 (67.36%)	
Risk aversion level			
1 (very low)	9 (56.25)	7 (43.75)	< 0.0001
2 (low)	42 (48.28)	45 (51.72)	
3 (neutral)	31 (31)	69 (69)	
4 (high)	31 (31)	69 (69)	
5 (very high)	7 (21.21)	26 (78.79)	
Entrepreneurial family member			
Yes	108 (41.22%)	154 (58.78%)	< 0.0001
No	12 (16.22%)	62 (83.78%)	

^*^Chi-squared test

Analysis results of the full model are reported in Table 4, using a multivariable logistic regression model. The odds ratio for females is 0.365 (*p* = 0.001), indicating that the probability of a female dentist reporting planning to enter the private sector (EI) on average is only about 36.5% as likely as a male respondent. The EI of dentists in the age group of 36–45 years and 46–60 years were about 14 times (*p* = 0.012) and 8.45 times (*p* = 0.066) respectively as likely as those in the age group of 20–25 years.

**Table 4:** Multivariate logistic regression analysis of Dentists' entrepreneurial intention and associated factors (Full model, with personality variables) (N = 336)

	Odds ratio (95% CI)	*P* value
Gender		
Male	1	
Female	0.365 (0.197–0.676)	0.001
Age (years)		
20–25	1	
26–35	2.915 (0.81–10.487)	0.101
36–45	14.205 (1.804–111.871)	0.012
46–60	8.45 (0.868–82.254)	0.066
Educational level		
Associate degree	1	
Bachelor's degree	0.36 (0.111–1.169)	0.089
Master's degree	0.221 (0.061–0.798)	0.021
Doctoral degree	0.118 (0.026–0.531)	0.005
Professional qualification		
Intern	1	
Resident	1.654 (0.52–5.264)	0.394
Attending dentist	7.812 (1.471–41.475)	0.016
Associate/chief dentists	9.857 (1.411–68.848)	0.021
Administration	0.827 (0.206–3.318)	0.789
Years of practice		
1 or less	1	
2–5	0.389 (0.154–0.982)	0.046
6–10	0.133 (0.027–0.664)	0.014
11 or more	0.015 (0.002–0.143)	< 0.0001
Number of employees at workplace hospital		
201 or more	1	
101–200	3.554 (1.09–11.588)	0.036
51–100	1.163 (0.426–3.179)	0.768
50 or less	2.398 (1.025–5.611)	0.044
Entrepreneurial behavior	3.602 (2.152–6.028)	< 0.0001
Risk aversion	0.702 (0.542–0.909)	0.007
Have entrepreneurial family member	3.672 (1.571–8.584)	0.003

Respondents with a higher Educational level are less likely to report EI, especially dentists with masters (OR = 0.221, *p* = 0.021) or doctoral degrees (OR = 0.118, *p* = 0.005). Regarding the professional qualification status of respondents, an attending dentist (OR = 7.812, *p* = 0.016) and associate/chief dentists (OR = 9.857, *p* = 0.021) have significantly much higher odds of reporting EI. Dentists in administration positions are not significantly more likely to report EI.

Meanwhile, years of practice are negatively associated with the EI. The odds ratios for dentists with 6–10 years and those with 11-years of work experience are 0.133 (*p* = 0.014) and 0.015 (*p* < 0.0001) respectively. The results suggest that dentists from mid-large hospitals (with 101–200 employees) (OR = 3.554, *p* = 0.036) and small public hospitals (with less than 50 employees) (OR = 2.398, *p* = 0.044) are on average significantly more likely than those in large hospitals (more than 200 employees) to report stronger EI, whereas the difference among those in medium-size hospitals (with 51–100 employees) are insignificant.

As reported in the bottom lines of Table 4, dentists with a higher level of entrepreneurial behaviors (OR = 3.602, p < 0.0001), or with an entrepreneur family member (OR = 3.672, *p* = 0.003), are significantly more likely to report EI. However, dentists with stronger risk aversion are less likely to report EI (OR = 0.702, *p* = 0.007).

## Discussion

As for the association between EI and demographic characteristics, the findings in this study are generally consistent with existing literature, but with some exceptions due to the knowledge-intensive feature of dentistry.

Female dentists in this study are found on average to be less likely than males to report having EI. This finding is consistent with existing literature [52–54]. The driving forces and pathways behind the gender difference are complicated [53] and may be due to various factors, such as social/cultural gender roles [55, 56], natural differences in personality and family responsibilities [57, 58].

The findings in the study indicate that age is a key factor with a strong association with dentists’ EI, and with the strongest effects among the group of 36–45 years old and remaining strong among the upper middle age group. Unlike general business startups with active innovations by the young generations [19–21], the professional qualification or criteria required by dentistry can only be obtained after years of education, practice, and experience.

A wide range of literature reports that education helps to increase EI through developing the natural entrepreneurial tendencies and enhancing the managerial ability of individuals [59]. However, this study finds that dentists in China with postgraduate education are much less likely to report having EI, when other potential determinant factors are controlled. This may be due to the fact that, during the past decades, dental education in China has been focusing on either the research and academic career, or a career path in prestigious hospitals, with no or very little entrepreneurial knowledge and training provided. Additionally, dentists with long years of postgraduate dental education have a greater opportunity cost for their career and social reputation if they take risks to engage in entrepreneurship [60].

A dentist’s professional qualification is another key factor with a strong positive association for EI. This finding can be explained by the fact that dentistry highly values professional qualification, which is especially necessary for the private sector if they want to obtain accreditation or to attract patients [61, 62].

The findings in this study indicate that years of practice are negatively associated with EI among dentists in China, holding other factors constant. Given a small group of literature reporting the decline in self-employment among near-retirees [63], most literature studying general business reports positive associations between working experience and EI [64–66]. Actually, the findings in this study have reasonably reflected the professional feature of dentistry. First, without the sufficient professional qualification for dentistry, years of practice alone does not bring a career advantage in private sector practice. Second, the positive effects of entrepreneurial knowledge acquired through work experience have largely been reflected in the regression analysis by the variable of professional qualifications for dentists. In China, some senior-aged dentists received only a two-year college level education in the late 1980’s and currently can only attain a relatively low level of professional qualification despite their many years of working.

The results in the full model in Table 4 indicate that dentists in mid-large hospitals (with 101–200 employees) and the small hospitals (with less than 50 employees) have a much higher level of EI. The overall professional satisfaction of a dentist’s career in China is most associated with factors such as respect, delivery of care, income, and patient relations [67]. The large hospitals in China, those with top tertiary connections, are always regarded with strong respect and supported with various types of social and financial resources. Meanwhile, small hospitals are on the other side of the spectrum. Hence, the career opportunity costs of entrepreneurial activities for the dentists from small hospitals are lower than for those in large hospitals. Dentists in mid-large hospitals may have a strong educational background and high quality skills too, but hold less prestigious career positions compared to those in top large hospitals. Taking these factors together into consideration, they may have a strong EI to start private practice to promote their career.

This study found that dentists’ entrepreneurial behavior levels had a significant positive association with their EI. These results are consistent with existing literature [35, 68]. Entrepreneurial behavior features actually reflect a person’s personality traits that indicate the possibility of becoming a leader, traits such as being proactive and innovative, and strong teamwork skills [14]. This finding also suggested that dentists with strong leadership skills or management skills in the public hospitals of China have high probabilities to leave for their own entrepreneurial activities.

This study found highly significant negative association between risk aversion and EI among dentists in China’s public hospitals. Risk aversion may indirectly reduce the possibility of EI through the channels of entrepreneurial skills and self-efficacy [60]. While dentists in China are more sophisticated and socially experienced than dental students studied [60], they actually may be even more risk-averse due to the larger social and economic opportunity cost in terms of career path, reputation and family economic responsibilities to be considered.

This study found a strong positive association between an individual’s EI and their family members or relatives involvement in dentistry entrepreneurial activities. This result is consistent with literature [22, 23]. Further, active involvement in a social network may help to identify and obtain various resources needed for a start-up [36, 69].

## Limitation

This study is a cross sectional survey, hence it has the limitations inherent in this research method. First, there may be non-random sampling errors and measurement errors during the process of the survey. However, these errors are not major concerns in this study, because precautious actions and sampling process management were performed to minimize these errors, though this is no guarantee of complete elimination.

Second, this study is based on 336 observations of dentists in Guangdong Province of China, which is among the most developed area in China. Hence, caution is necessary when generalizing the findings to the national level.

Third, due to the survey research design using observatory data, this study may be subject to the survival bias. Dentists who are very good at entrepreneurial activities, or with strong skills may have left the public hospitals to start their private practice, and as a consequence, only those with low EI and skills remain working in the public hospitals. Hence, the analysis was limited to an associative relationship, rather than being interpreted as causal effects.

Forth, the analysis about EI may be subject to the omitted variable bias. There are actually various factors (e.g., personality characteristics and social/culture environment) affecting a person’s EI and these factors may also have complicated interaction effects on each other. However, it is impossible to include all potential influential factors in the estimation model.

## Conclusions

Analyzing the survey results of 336 dentists from public hospitals in five major cities in Guangdong Province (China), this study found that, within the knowledge-intensive industry of dentistry, dentists’ entrepreneurial behaviors have their own features. Accumulation of practical skills during their career, as implied by age, professional qualifications and leadership skills, help to promote EI among dentists, whereas an academic oriented education degree per se does not promote EI. Post-graduate education and years of practice per se are not directly positively associated with a higher level of EI among dentists. The finding that dentists in mid-large and small hospitals in China are more likely to report EI is counter to the traditional perception, one that is biased toward the dentists in top large hospitals. Additionally, female dentists have a lower level of EI, suggesting more social supports or self-efficacy are necessary to promote female dentists’ entrepreneurship and to help them to choose optimal career path.

## Supplementary information

**Additional file 1.** Questionnaire for survey about the entrepreneurial intention of dentists in public hospitals in major cities in Guangdong (South China).

## Data Availability

The datasets used and analyzed in the current study are available from the corresponding author upon request.

## Abbreviation

EI: Entrepreneurial intention
