# Supplementary material for: Dentists’ entrepreneurial intention and associated factors in public hospitals in major cities in Guangdong (South China): a cross-sectional study
Source: BMC Oral Health. 2020 Nov 23;20:334. doi: 10.1186/s12903-020-01331-z (PMC7685661; doi:10.1186/s12903-020-01331-z)
Supplement: Supplementary file 1 — Additional file 1. Questionnaire for survey about the entrepreneurial intention of dentists in public hospitals in major cities in Guangdong (South China). [file 12903_2020_1331_MOESM1_ESM.docx]

**Additional files**

**Questionnaire for survey about the entrepreneurial intention of Chinese dentists**

Dear Madam/Sir:

This survey is a study on the entrepreneurial intention of Chinese dentists conducted by Macau University of Science and Technology. The purpose of this survey is to support and promote the success of dentists’ entrepreneurial ventures. We promise to keep your answers strictly confidential under any condition. Your response to this survey indicates that you have read and agreed with our disclosure. This questionnaire takes only 5 minutes to complete.

Sincere thanks for your cooperation!

Macau University of Science and Technology

**Part 1 basic situation survey** (please tick the corresponding option)

(1) Your gender:

A. male B. female

(2) Your age:

A. 20-25 B. 26-35 C. 36-45 D 46-60

(3) Your Educational level:

A. Associate B. Bachelor C. Master D. Doctor

(4) Your Professional qualification:

A. Intern

B. Resident

C. Attending dentist

D. Associate/ Chief dentists E. Administration

(5) The number of employees at your workplace hospital:

A. 50 or less

B. 51 to 100

C. 101 to 200

D. 201 or more

(6) Your years of practice:

A. Less than 1 year

B. 2-5 years

C. 6-10 years

D. Over 11 years

(7) Do you have family/relative members who engaged in entrepreneurship?

 ⬜A. Yes ⬜B. No

(8). It is likely that I will start and run my business in dentistry in the near future.

| 1 | 2 | 3 | 4 | 5 |
| --- | --- | --- | --- | --- |
| very unlikely | unlikely | neutral | likely | most likely |
| ⬜ | ⬜ | ⬜ | ⬜ | ⬜ |

**Part 2 Please answer the following questions according to your personal situation.**

（Strongly disagree= 1, Disagree=2, Undecided=3, Agree=4, Strongly Agree=5）

| There are no correct or incorrect answers to the following questions. If the answer is close to your personal situation, please type "V" in the place. | 1 | 2 | 3 | 4 | 5 |
| --- | --- | --- | --- | --- | --- |
| 1. The failure of starting a private business will have a negative impact on my future career. | ⬜ | ⬜ | ⬜ | ⬜ | ⬜ |
| 2. I encourage others to take the initiative for their own ideas. | ⬜ | ⬜ | ⬜ | ⬜ | ⬜ |
| 3. I inspire others to think about their work in new and stimulating ways. | ⬜ | ⬜ | ⬜ | ⬜ | ⬜ |
| 4. I devote time to helping others find ways to improve our products and services. | ⬜ | ⬜ | ⬜ | ⬜ | ⬜ |
| 5. I vividly describe how things could be in the future and what is needed to get us there. | ⬜ | ⬜ | ⬜ | ⬜ | ⬜ |
| 6. I get people to rally together to meet a challenge. | ⬜ | ⬜ | ⬜ | ⬜ | ⬜ |
| 7. I create an environment where people get excited about making improvement. | ⬜ | ⬜ | ⬜ | ⬜ | ⬜ |
